# Constitutive Expression of *Aechmea fasciata SPL14 (AfSPL14)* Accelerates Flowering and Changes the Plant Architecture in *Arabidopsis*

**DOI:** 10.3390/ijms19072085

**Published:** 2018-07-18

**Authors:** Ming Lei, Zhi-ying Li, Jia-bin Wang, Yun-liu Fu, Meng-fei Ao, Li Xu

**Affiliations:** 1 Institute of Tropical Crops Genetic Resources, Chinese Academy of Tropical Agricultural Sciences, Danzhou 571737, China; leiming_catas@126.com (M.L.); xllizhiying@vip.163.com (Z.-y.L.); jiabinwangfuhu@sina.com (J.-b.W.); fyljj_2007@126.com (Y.-l.F.); 17889981612@163.com (M.-f.A.); 2 Key Laboratory of Crop Gene Resources and Germplasm Enhancement in Southern China, Danzhou 571737, China; 3 Key Laboratory of Tropical Crops Germplasm Resources Genetic Improvement and Innovation, Danzhou 571737, China; 4 Mid Tropical Crop Gene Bank of National Crop Resources, Danzhou 571737, China

**Keywords:** *Aechmea fasciata*, squamosa promoter binding protein-like, flowering time, plant architecture, bromeliad

## Abstract

Variations in flowering time and plant architecture have a crucial impact on crop biomass and yield, as well as the aesthetic value of ornamental plants. *Aechmea fasciata*, a member of the Bromeliaceae family, is a bromeliad variety that is commonly cultivated worldwide. Here, we report the characterization of *AfSPL14*, a squamosa promoter binding protein-like gene in *A. fasciata*. *AfSPL14* was predominantly expressed in the young vegetative organs of adult plants. The expression of *AfSPL14* could be upregulated within 1 h by exogenous ethephon treatment. The constitutive expression of *AfSPL14* in *Arabidopsis thaliana* caused early flowering and variations in plant architecture, including smaller rosette leaves and thicker and increased numbers of main inflorescences. Our findings suggest that AfSPL14 may help facilitate the molecular breeding of *A. fasciata*, other ornamental and edible bromeliads (e.g., pineapple), and even cereal crops.

## 1. Introduction

The squamosa promoter binding protein (SBP)-like (SPL) proteins are plant-specific transcription factors (TFs) that play essential roles in the regulation networks of plant growth and development [1]. The genes encoding SPL proteins were first identified in snapdragon (*Antirrhinum majus*), and were then found in almost all other green plants [2,3,4,5]. All SPL proteins contain a highly conserved DNA-binding domain termed the SBP domain, which consists of approximately 76 amino acid residues and features two zinc-binding sites and a bipartite nuclear localization signal (NLS) [6]. Many studies of various species have revealed the diverse functions of SPLs, which are involved in a broad range of important biological processes including the leaf development [7,8,9,10], embryonic development [11], fertility controlling [12,13], copper homeostasis [14,15], as well as the biosynthesis of phenylpropanoids and sesquiterpene. In addition to affecting these developmental aspects, several SPL factors which can be regulated by miR156, an evolutionary highly conserved microRNA (miRNA), also play crucial roles in the control of flowering time. The overexpression of *AtSPL3*, *AtSPL4*, *AtSPL5*, *AtSPL9*, *AtSPL15,* and *OsSPL16* can significantly promote flowering [3,9,16,17,18,19]. AtSPL9, together with AtSPL3 and the AtSPL2/10/11 group promote the floral meristem identity by directly regulating the same or different target genes [9,19]. Interestingly, compared with the positive regulation of accelerated flowering by the SPLs described above, AtSPL14 appears to be a negative regulator of vegetative-phase changes and floral transitions [20]. Another function of miR156-regulated SPL factors is in plant architecture formation and yield. *Teosinte Glume Architecture* (*TGA1*), an *SPL* gene, is responsible for the liberation of the kernel during domestication and evolution in *Z. mays* [21]. In *Triticum aestivum*, TaSPL3/17 play important roles in reducing the number of tillers and the outgrowth rate of axillary buds [22]. Two SPL homologs, TaSPL20 and TaSPL21, together reduce plant height and increase the thousand-grain weight [23]. In switchgrass (*Panicum virgatum*), miR156-regulated SPL4 suppresses the formation of both aerial and basal buds and controls the shoot architecture [24]. In *O. sativa*, higher expression of *OsSPL14* can reduce the tiller number, increase the lodging resistance, promote panicle branching and enhance grain yields [25,26]. The multifaceted functions of SPLs demonstrate complex and interesting regulation networks underlying plant lifestyles.

Bromeliaceae is one of the most morphologically diverse families and is widely distributed in tropical and subtropical areas [27]. Although certain cultivated species of bromeliads are appreciated for their edible fruits (e.g., pineapple: *Ananas comosus*) or medicinal properties (e.g., *Bromelia antiacantha*), the vast majority are appreciated for their ornamental value [28]. However, the unsynchronized natural flowering time of cultivated bromeliads always results in increased cultivation and harvesting costs and decreased economic value of fruits and ornamental flowers [29]. To date, several efforts were made to uncover the mechanism of flowering of bromeliads induced by age, photoperiod, autonomous and exogenous ethylene, or ethephon [29,30,31,32,33], but the precise molecular mechanism remained unknown.

Here, we characterized the *SPL* gene *AfSPL14*, from *Aechmea fasciata*, a popular ornamental flowering bromeliad. Phylogenetic analyses showed that AfSPL14 is closely related to OsSPL17, OsSPL14, AtSPL9, and AtSPL15. Furthermore, the expression of *AfSPL14* transcripts responded to plant age and exogenous ethephon treatment. The constitutive expression of *AfSPL14* in *Arabidopsis* promotes branching and accelerates flowering under long-day (LD) conditions. These results suggested that AfSPL14, a TF of the SPL family, might be involved in the process of flowering and in plant architecture variations of *A. fasciata*.

## 2. Results

### 2.1. Isolation and Sequence Analysis of AfSPL14 in A. fasciata

The *SPL* cDNA was isolated using the rapid amplification of cDNA ends (RACE) technique, and then named *AfSPL14*. The cDNA of *AfSPL14* was 1504-bp long and presented a 123-bp 5′ untranslated region (UTR), a 331-bp 3′ UTR, and a 1050-bp open reading frame (ORF), which was predicted to encode a 349-amino acid protein with a molecular weight (MW) and an isoelectric point (pI) of 37.53 kDa and 9.08, respectively.

**Figure 1:**
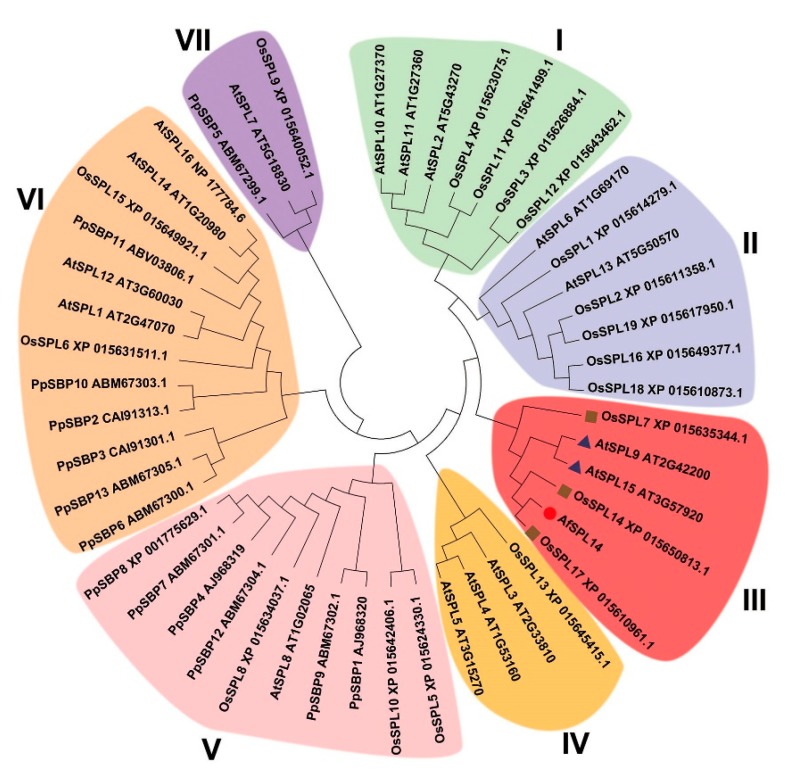
Phylogenetic analysis of AfSPL14 and SPLs of *Arabidopsis*, *O. sativa* and *P. patens* based on the conserved SBP domains. The unrooted tree was created using the neighbor-joining method with 1000 bootstrap replicates with 13 SBPs of *P. patens*, 16 SPLs of *Arabidopsis*, and 19 SPLs of *O. sativa*. The sequences of all these SBP domains are listed in Table S1.

To investigate the evolutionary relationships between the AfSPL14 and SPL proteins of other species, a phylogenetic tree was constructed using the neighbor-joining method with 1000 bootstrap replicates with 13 SBPs of *Physcomitrella patens*, 16 SPLs of *Arabidopsis*, and 19 SPLs of *O. sativa* (Table S1). Because the alignment of the full-length protein sequences showed no consensus sequences except for SBP domains (data not shown), only the highly-conserved SBP domains were used for the phylogenetic analysis. The unrooted phylogenetic tree classified all SBP domains into seven groups (I-VII), and AfSPL14 was clustered into group III, with AtSPL9, AtSPL15, OsSPL7, OsSPL14, and OsSPL17; however, this group did not contain SBP domains of *P. patens* (Figure 1), which was similar to the results obtained by others [34]. The fact that AfSPL14 has been classified with three SPL genes in *O. sativa* and two in *Arabidopsis* suggests that the SBP domains of these six SPLs might have undergone species-specific evolutionary processes after speciation.

**Figure 2:**
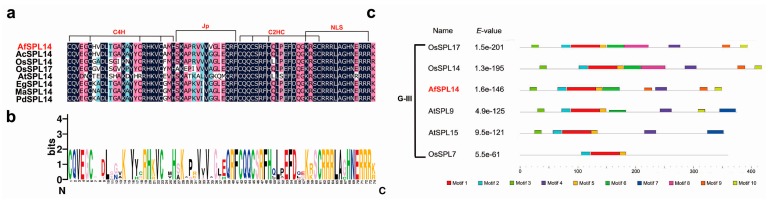
Sequence alignment and logo view of variable SBP domains, and putative motifs of variable SPLs in group III. (**a**) Multiple alignment of the SBP domains using DNAMAN software. The four conserved motifs, which include two zinc finger-like structures (C4H, C2HC), Jp and NLS, are indicated; (**b**) Sequence logo view of the consensus SBP domains. The overall height of the stack and the height of each letter represent the sequence conservation at that position and the relative frequency of the corresponding amino acid at that position, respectively; (**c**) putative motifs of variable SPLs in group III identified by MEME software online (http://meme-suite.org/tools/meme). The color boxes represent different putative motifs for which the sequences are listed in Table S2 online. G-III indicates group III from Figure 1.

The multiple sequence alignment of the AfSPL14 protein with SPL homologs of other species indicated that the SBP domain was highly conserved among the species (Figure 2a). All SBP domains could be divided into four motifs, which were zinc finger-like structure 1 (Zn1), Zn2, joint peptide (Jp) of Zn1 and Zn2, and NLS. The first zinc finger was C4H, and the second zinc finger-like structure was C2HC. The Jp plays a crucial role in modifying the protein-DNA interaction process [6], and was also highly conserved in all aligned sequences (Figure 2a). In addition, the bipartite NLS motif, which partially overlapped Zn2, was highly conserved (Figure 2a,b).

To further examine conserved sequences other than the SBP domain, the online Multiple EM for Motif Elicitation (MEME) tool was used to identify putative motifs in the SPL proteins in group III [35]. As shown in Figure 2c, all members of group III contained motifs 1, 2, and 5, and these motifs had similar distributions. Actually, motif 2 belongs to C4H, and motif 5 belongs to NLS; motif 1 contains Jp, C2HC, and partials of C4H and NLS (Figure 2c, Table S2). Compared with OsSPL14 and OsSPL17, which contained all motifs except motif 7, AfSPL14 had a similar motif distribution but lacked motifs 7 and 8 (Figure 2c). These results suggested that AfSPL14 might have conserved functions with OsSPL14 and OsSPL17.

**Figure 3:**
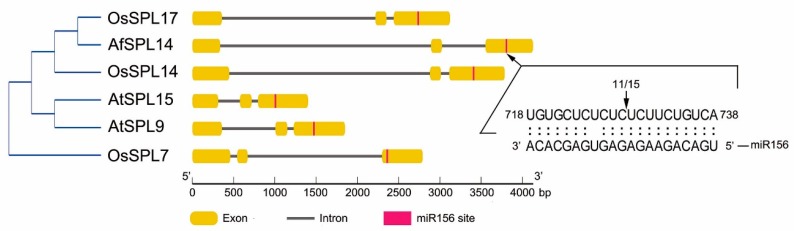
Exon-intron structures of *SPL* genes in group III from Figure 2 and AfmiR156 cleavage site in *AfSPL14* determined by 5′ RLM-RACE. For the determination of the AfmiR156 cleavage site in *AfSPL14*, 15 clones were selected randomly for sequencing, and 11 of them were cleaved in the position indicated by the arrow towards the base interval of *AfSPL14* RNA sequence.

Exon-intron organization of all members of group III genes were generated based on genome sequences and the corresponding CDSs (Figure 3). As shown in Figure 3, each member of these genes had two introns and three exons; thus, they shared a similar exon-intron composition. All members of rice and *Arabidopsis* in group III were targets of miR156 [9,36], and a putative miR156 target site was also observed in *AfSPL14* (Figure 3). The consistency of the motif investigation, exon-intron organization, and phylogenetic analysis indicates putative similarities in functional regions and sites among the genes in group III.

### 2.2. AfSPL14 Was a Target of miR156 of A. fasciata (AfmiR156)

As all members of rice and *Arabidopsis* in group III were targets of miR156, there was also a putative miR156 target site in *AfSPL14* (Figure 3). To test whether the mRNA of *AfSPL14* was indeed targeted for degradation and was cleaved at the predicted position by AfmiR156, 5′ RNA ligase mediated rapid amplification of cDNA ends (RLM-RACE) was carried out to map the 5′ terminus of the cleavage fragment. DNA sequencing results of the amplified product demonstrated that *AfSPL14* could be indeed cleaved by AfmiR156 (Figure 3).

### 2.3. Transcript Profiling of AfSPL14 in A. fasciata

**Figure 4:**
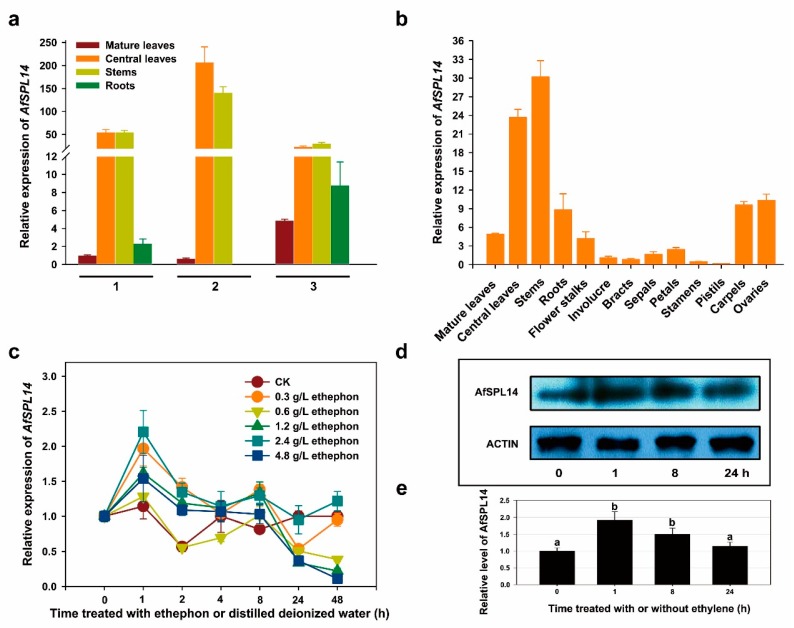
Expression of *AfSPL14* transcripts in various tissues of *A. fasciata* and immunoblot analysis of AfSPL14 in central leaves treated with or without ethephon. (**a**) Expression level of *AfSPL14* transcripts in various tissues of juvenile and adult plants. (1) juvenile plants; (2) adult plants prior to flower bud differentiation; (3) 39-DAF flowering adult plants. Samples were collected at 10:00 am. (**b**) Expression level of *AfSPL14* transcripts in the vegetative and reproductive organs of 39-DAF flowering adult plants. Samples were collected at 10:00 am. (**c**) Expression level of *AfSPL14* transcripts in the central leaves of *A. fasciata* in response to exogenous ethephon treatment at different concentrations for different time. In the panels, 0, 1, 2, 4, and 8 h represents the samples collected at 10:00, 11:00, 12:00, 14:00, and 18:00, respectively; 24 h and 48 h represent the treated samples collected at 10:00 am at the next day and the next two days, respectively. For CK, 10 mL of distilled deionized H_2_O was poured into the cylinder shapes of *A. fasciata*. 0 h represents the samples treated without ethephon or distilled deionized H_2_O. (**d**) Immunoblot analysis of the AfSPL14 protein level in the central leaves of *A. fasciata* treated with 10 mL of 0.6 g·L^−1^ exogenous ethephon for 1, 8, and 24 h, or without ethephon (0 h). The total proteins were separated using SDS-PAGE, and the transferred proteins were then probed with a rabbit polyclonal AfSPL14 antibody or a rabbit polyclonal Actin antibody, respectively. (**e**) Relative level of AfSPL14 protein in the central leaves of *A. fasciata* treated with 10 mL of 0.6 g·L^−1^ exogenous ethephon for 1, 8 and 24 h, or without ethephon (0 h). Three independent experiments were performed, the values are shown as the means and error bars indicate the standard deviation (*n* = 3). ANOVA was conducted, and means were separated by DNMRT.

To gain insights into the role of AfSPL14 in *A. fasciata*, we determined the gene’s expression profiles in various organs at different developmental stages via reverse transcription followed by quantitative real-time PCR (RT-qPCR). The transcripts of *AfSPL14* could be detected in almost all tested tissues except the roots of the adult plant prior to flower bud differentiation (Figure 4a). *AfSPL14* mRNA was more abundant in the central leaves and stems regardless of the developmental stage (Figure 4a,b). The accumulation of *AfSPL14* transcripts in the central leaves and stems showed significant changes during development, with the highest level observed in adult plants prior to flower bud differentiation, a relatively lower level observed in juvenile plants, and the lowest level observed in 39-day-after-flowering (DAF) adult plants (Figure 4a,b), suggesting that AfSPL14 might be involved in phase transitions.

### 2.4. Response to Exogenous Ethephon Treatment

To induce bromeliad flowering, ethylene or ethephon is widely used [29]. In fact, flowering induction by ethylene or ethephon is age-dependent. Plants of *A. comosus* which were somewhat less than about 1.0 kg fresh weight in subtropical regions respond only minimally to ethylene or ethephon [29]. Similar to ‘Smooth Cayenne’ and other variations of *A. comosus*, adult plants (but not juveniles) of *A. fasciata* could be induced by ethephon. Our previous investigation also showed that above 96% of 12-month-old adult plants could be induced to flower by 10 mL of exogenous ethephon treatment at 0.6 g·L^−1^ within two weeks, but that none of 6-month-old juvenile plants flowered under the same condition [37]. Here, we investigated the possible response of *AfSPL14* in adult plants of *A. fasciata* to exogenous ethephon treatment at different concentrations. As shown in Figure 4c, the expression of *AfSPL14* transcripts in the central leaves of adult plants prior to flower bud differentiation increased transiently after treatment for 1 h. Interestingly, a rapid decrease of the expression level of *AfSPL14* transcripts was observed after treatment for 2 h, almost reaching lower levels than in control plants after 24 h (Figure 4c), suggesting the remarkable effect of ethylene on the expression of *AfSPL14*.

To investigate the effect of ethylene on the level of AfSPL14 protein, we extracted the total proteins from the central leaves of *A. fasciata* treated with or without 10 mL of 0.6 g·L^−1^ ethephon. An immunoblot analysis was performed using a specific antibody against the AfSPL14 protein (Figure 4d). The level of AfSPL14 after treatment for 1 h was ~200% higher than the level in the untreated central leaves (Figure 4d,e). Consistent with the changes in the relative expression of *AfSPL14* mRNA, the level of AfSPL14 also gradually decreased after continuous treatment for 8 and 24 h (Figure 4d,e).

### 2.5. AfSPL14 Does Not Exhibit Transactivation Activity in Yeast

**Figure 5:**
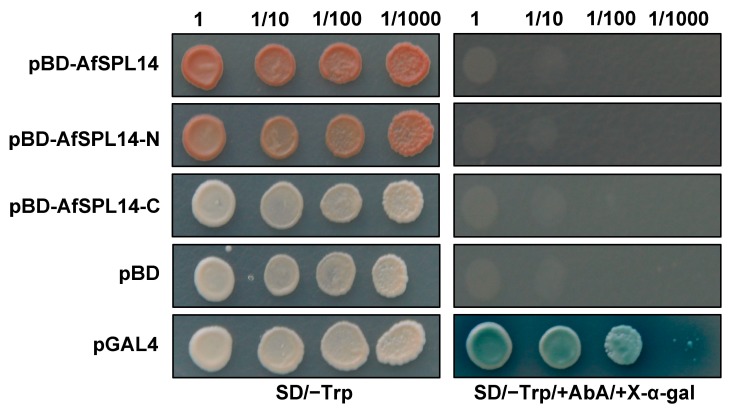
Transactivation activity assay of AfSPL14 in yeast cells. The pGBKT7 (pBD) vectors were fused with the full-length of AfSPL14 (pBD-AfSPL14), the N terminus of AfSPL14 (pBD-AfSPL14-N) and the C terminus of AfSPL14 (pBD-AfSPL14-C), respectively. Each kind of these constructs was then transformed into Y2HGold cells which contained the reporter genes *AUR1-C* and *MEL1*. pBD and pGAL4 plasmids were transformed into Y2HGold cells and used as negative and positive controls, respectively. Yeast clones containing the right constructs grew on SD/−Trp medium at dilutions of 1, 1/10, 1, 100, and 1/1000 for three to five days, and were then transferred onto SD/−Trp/+AbA/+X-α-Gal medium for continuous growth for three further days to test their transactivation activities. SD: synthetic dropout; AbA: Aureobasidin A; SD/−Trp: SD medium without Trp; SD/−Trp/+AbA/+X-α-gal: SD medium without Trp, but with 40 mg/L X-α-gal and 200 μg/L AbA.

To test whether AfSPL14 is a transcriptional activator, the ORF (1-349 amino acids), the N terminus containing the SBP domain (1–141 amino acids) (AfSPL14N), and the C terminus (142–349 amino acids) (AfSPL14C) of AfSPL14 were fused with the GAL4 binding domain carried by the pGBKT7 (pBD) vector, respectively. The expression vectors pBD-AfSPL14, pBD-AfSPL14N, and pBD-AfSPL14C were then transformed into the yeast strain Y2HGold carrying the dual reporter genes AUR1-C and MEL1, respectively. As shown in Figure 5, similar to the negative control pBD, but not the positive control pGAL4, all the yeast cells carrying the three tested vectors could not grow on a medium containing SD/−Trp/+AbA/+X-α-Gal, indicating that AfSPL14 could not activate the transcription of the dual reporter genes in yeast.

### 2.6. Constitutive Expression of AfSPL14 in Arabidopsis

**Figure 6:**
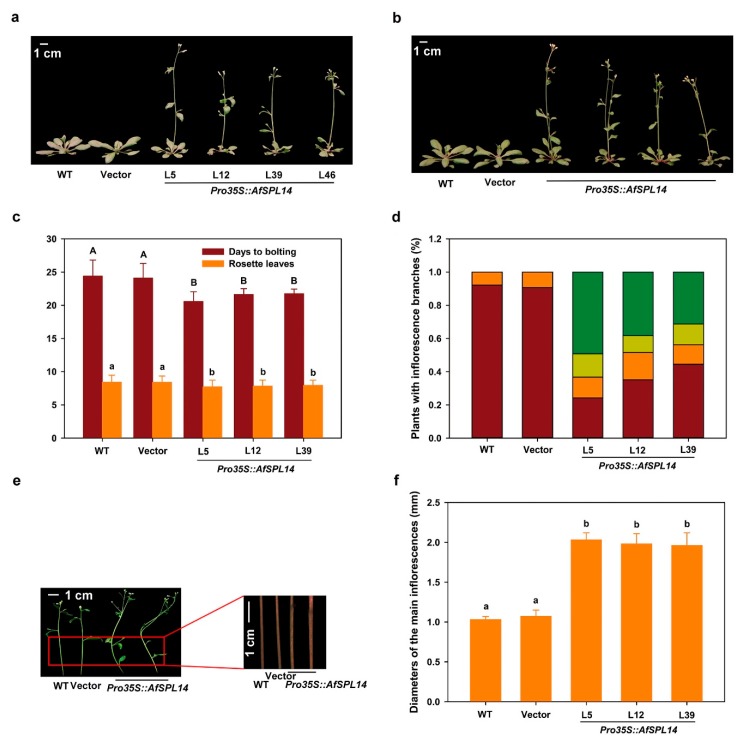
Phenotype analysis of *Pro35S::AfSPL14* transgenic plants. (**a**) Flowering *Pro35S::AfSPL14* transgenic plants shown next to WT and WT transformed with the empty vector (Vector) under LD conditions. L5, L12, L39, and L46 indicate the different lines. (**b**) Flowering *Pro35S::AfSPL14* transgenic plants that had two main inflorescences under LD conditions. (**c**) Days and number of rosette leaves to bolting of the WT, Vector and *Pro35S::AfSPL14* transgenic plants grown under LD conditions. Values are the means ± standard deviation. Seventy-nine plants were scored for each line. Difference letters indicate statistical differences. (**d**) Percentages of plants which have two main inflorescences grown under LD conditions. The number of plants with the second main stem developed from the base (orange), the node (ginger), and the non-node (dark green) position of the main inflorescences and plants with only one inflorescence (dark red) was calculated. One hundred and twenty-eight 38-day-old, long-day-grown plants and ninety-six 55-day-old, short-day-grown plants were counted for each line. (**e**) Bending and thicker main inflorescences of *Pro35S::AfSPL14* transgenic plants under LD conditions. (**f**) The diameter of main inflorescences of WT, Vector and transgenic plants. Forty-eight 38-day-old, long-day-grown plants were counted for each line. The diameter of the positions which were 5 cm distance from the basal of the main inflorescences was measured. ANOVA was conducted, and means were separated by DNMRT.

To assess the function of AfSPL14 in flowering, we induced the ectopic expression of *AfSPL14* with the 35S CaMV promoter (*Pro35S::AfSPL14*) in *Arabidopsis* ecotype Columbia (Col-0) (WT) (Figure S1). Under LD conditions, the flowering time of *Pro35S::AfSPL14* transgenic plants was significantly earlier (*p* = 6.26 × 10^−8^) than that of the WT and the WT transformed with the empty vector (Vector) (Figure 6a–c). Although the difference of the number of rosette leaves was minor between the *Pro35S::AfSPL14* transgenic plants and WT, the statistical analysis indicated that the number was significantly lower (*p* = 4.69 × 10^−5^) in the *Pro35S::AfSPL14* transgenic plants (Figure 6c). The *Pro35S::AfSPL14* transgenic lines were also smaller than the WT (Figure 6a–c). In addition, the *Pro35S::AfSPL14* transgenic plants showed morphological changes in the reproductive phase. A comparison between the WT and Vector, which only has one main inflorescence per plant, showed that a majority of the transformants of *Pro35S::AfSPL14* developed two main inflorescences (Figure 6b,d). Interestingly, a second inflorescence could be developed from the base of the main inflorescence, and it could also develop from the node of the main inflorescence or even be divided randomly from the non-node position of the main inflorescence (Figure 6b,d). Another change in the reproductive phase was the thickening of the main inflorescence in the *Pro35S::AfSPL14* transgenic plants compared with that of the WT (Figure 6e,f).

**Figure 7:**
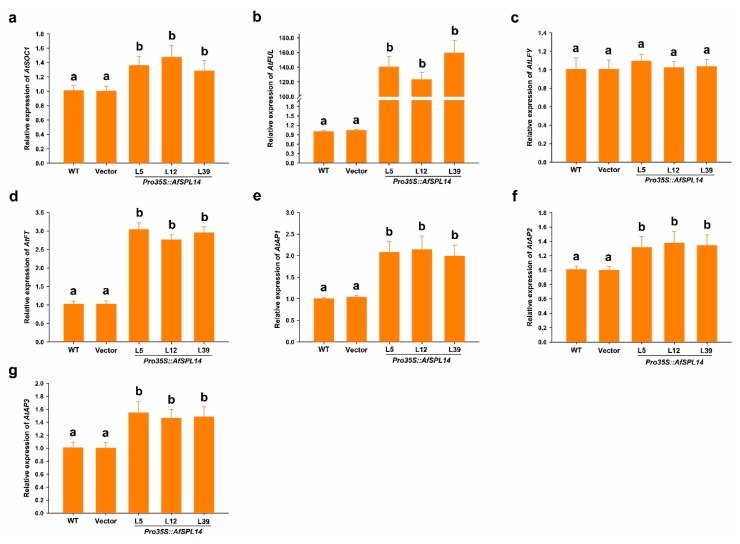
RT-qPCR analysis of flowering related genes at the shoot apex of WT, Vector and *Pro35S::AfSPL14* transgenic plants. Relative expression of three flowering promoting genes, *SUPPRESSOR OF OVEREXPRESSION OF CONSTANS 1* (*SOC1*) (**a**), *FRUITFULL* (*FUL*) (**b**), *LEAFY* (**c**), and one florigen *Flowering Locus T* (*FT*) (**d**), and three flowering organ identify genes, *APETALA1* (*AP1*) (**e**), *AP2* (**f**) and *AP3* (**g**) was performed. Fourteen-day-old long-day-grown seedlings were used. Three biological replicates and three technical replicates were performed. Transcript levels were normalized using *AtACTB* gene as a reference. All primers used here are listed in Table S3 online. ANOVA was conducted, and means were separated by DNMRT.

To further confirm whether the expression of *AfSPL14* in the *Pro35S::AfSPL14* transgenic plants altered the expression of downstream flowering genes, RT-qPCR analysis was performed with the *Arabidopsis* shoot apices grown under LD conditions as materials. The *Arabidopsis* shoot apices were harvested from the central parts of *Arabidopsis* seedlings, and contain the youngest rosette leaves. As expected, compared to WT, the expression level of the genes *SUPPRESSOR OF OVEREXPRESSION OF CONSTANS 1* (*SOC1*), *FRUITFULL* (*FUL*) and *APETALA1* (*AP1*), which encode floral inductive factors, was substantially upregulated at the shoot apex of *Pro35S::AfSPL14* transgenic plants (Figure 7a,b,e). However, the expression level of another gene encoding plant-specific transcription factor LEAFY (LFY), which is also a positive regulator inducing flowering at the shoot apex, showed no clear difference between WT and *Pro35S::AfSPL14* transgenic plants (Figure 7c). The expression level of *Flowering Locus T* (*FT*), an integrator of flowering pathways and defined as a florigen, was also considerably upregulated at the shoot apex of *Pro35S::AfSPL14* transgenic plants (Figure 7d). In addition, the expression of floral organ identity genes, such as *AtAP2* and *AtAP3*, was also upregulated (Figure 7f,g).

## 3. Discussion

SPL proteins are plant-specific TFs, and have been reported in many plants, including *Antirrhinum majus* [2], *Arabidopsis thaliana* [3], *Chlamydomonas* [14], *O. sativa* [36], *P. patens* [5], tomato [38], *Triticum aestivum* [39], *Castor Bean* [40], *Prunus mume* [41], *Citrus* [42], pepper [43], Petunia [44], *Brassica napus* [45] and *Chrysanthemum* [46]. In the present study, we identified the *SPL* gene *AfSPL14* in *A. fasciata*, an economically valuable, short-day ornamental plant that exhibits crassulacean acid metabolism (CAM). We discussed the correlation between this gene and the plant hormone ethylene, which has been widely used to induce flowering of members in numbers of the Bromeliaceae family. We also suggested that AfSPL14 was a putative flowering inducer and ideal plant architecture generator, based on its heterologous constitutive expression in *Arabidopsis*.

Compared with many other plant species in which the role of ethylene in the regulation of flowering appears complicated, in a majority of bromeliads including pineapple and *A. fasciata*, flowering can be triggered by a small burst of ethylene production in the meristem in response to exogenous ethylene or ethephon treatment [30,33]. In previous studies, several ethylene biosynthesis, signaling and responsive genes were identified and characterized [30,31,32,33,47]. Here, we found that exogenous ethephon induced the expression of *AfSPL14* transcripts rapidly and dramatically within 1 h (Figure 4c). Interestingly, the expression level of *AfSPL14* transcripts gradually declined after continuous treatment for 8 h (Figure 4c). In fact, several *SPLs* in some other species also could be transiently upregulated and then downregulated by ethylene, for example, *MdSBP20* and *MdSBP27* in the leaves of apple (*Malus × domestica* Borkh.) cv. ‘Fuji’34, and *SPL7* and *SPL9* in the fruit of *Cavendish banana* [48]. A more precise identification of the changes in the translational level of AfSPL14 in response to the exogenous ethephon treatment showed a consistence with the changes at the transcriptional level. After treatment for 1 h, the expression of the AfSPL14 protein was also dramatically induced to a higher level compared with that in the untreated central leaves (Figure 4d,e). However, after treatment for 8 h and 24 h, the amount of AfSPL14 also decreased gradually (Figure 4d,e). Furthermore, three 5′-ATGTA-3′ core sequences were enclosed in the nearly 3000-bp-length promoter sequence of *AfSPL14* promoter (Figure S2). The 5′-ATGTA-3′ core sequence might interact with ethylene insensitive 3 (EIN3), a crucial factor in the ethylene signaling pathway that could activate or inhibit the expression of downstream genes at the transcriptional level. Further investigation should be performed regarding the regulation of *AfSPL14* by exogenous ethephon at the transcriptional and post-transcriptional levels.

Previous studies of the molecular regulation of the model species *Arabidopsis* have identified at least five genetic pathways relevant to flowering, namely: the photoperiod, vernalization, gibberellic acid (GA), and the autonomous and aging pathways [49]. During this process, at least 180 genes were involved [50]. *SPLs* are indispensable among these genes, and are involved in several signaling pathways. For example, AtSPL3 and AtASPL9 act independently of *FT*, and directly activate flower-promoting *MADS box* genes, thus defining a separate endogenous flowering pathway [18]. AtSPL9 could also acts upstream of *FT* and promotes *FT* expression [51,52]. AtSPL15 integrates the GA pathway and the aging pathway to promote flowering [53]. In addition, AtSPL3/4/5 link developmental aging and photoperiodic flowering [54]. Moreover, the enhancement of the miR156 site-mutated *OsSPL14* gene could also accelerate flowering [55]. Phylogenetic and motif analyses of AfSPL14 and the SPLs of *Arabidopsis* and OsSPL14 showed that the former was similar to AtASPL9, AtSPL15, and OsSPL14 (Figure 1, Figure 2 and Figure 3), implying a putative conserved function, such as, flowering promotion. Recently, an age-dependent flowering pathway was identified by the regulation of CmNF-YB8, a nuclear factor, through directly triggering miR156-SPL-regulated processes in the short day plant chrysanthemum (*Chrysanthemum morifolium*) [56]. The flowering of pineapple and *A. fasciata* is also age dependent, and the juvenile plants cannot flower naturally, even when treated with exogenous ethylene [30,33]. The expression of *AfSPL14* transcripts was higher in the central leaves and stems of adult plants prior to flower bud differentiation compared with that of the juvenile plants (Figure 4a). This fact is similar to the increasing pattern of accumulation of *AtSPL9* and *AtSPL15* in the meristem with age [9,17,53], and inconsistent with the expression profile of *AfAP2-1*, a putative flowering TF encoding gene identified in *A. fasciata* [33]. These results suggest that AfSPL14 might act positively in the juvenile-to-vegetative phase transition and flowering pathway regulated by developmental age.

The constitutive expression of *AfSPL14* in *Arabidopsis* significantly promoted flowering under LD conditions (Figure 6a–c), which was inconsistent with the flowering-delayed phenotype caused by the constitutive expression of *AfAP2-1* in *Arabidopsis* [33], thus suggesting that AfSPL14 is an activator of flowering integrator and floral inductive genes such as *AtFT*, *AtAP1*, *AtSOC1,* and *AtFUL* (Figure 7a,b,d,e). A previous study demonstrated that the overexpression of AtSPL3 could strongly induce *AtFUL*, but has a weaker effect, or no effect at all, on *AtSOC1* in the shoot apex in *Arabidopsis* [18]. Interestingly, compared with that of WT, the expression level of *AtFUL* at the shoot apex of *Pro35S::AfSPL14* transgenic plants was upregulated dramatically, while *AtSOC1* was slightly induced (Figure 7a,b), suggesting that similar to AtSPL3, AfSPL14 might also induce flowering via an endogenous pathway.

Phylogenetic and motif analyses of AfSPL14 with variable SPLs suggested that it was closer and more similar to OsSPL14 than to AtSPL9 and AtSPL15 (Figure 2 and Figure 3); this is consistent with the evolutionary distances among *A. fasciata*, rice and *Arabidopsis*. In addition, the genes have similar exon-intron structures (Figure 3). Higher expression of *OsSPL14* could reduce the tiller number, increase the lodging resistance, promote panicle branching, and enhance the grain yield [25,26]. Importantly, in addition to the acceleration of flowering, the constitutive expression of *AfSPL14* in *Arabidopsis* also promotes the number of main inflorescences and produces thicker and sturdier culms (Figure 6a–f). However, we did not find the transactivator activity of AfSPL14 in yeast cells (Figure 5), in opposition to the results reported on OsSPL14 [26]. These results suggested functional conservation and diversification in AfSPL14 and OsSPL14. Interestingly, the repression of *AtSPL10* caused reduced apical dominance, and increased the number of main inflorescences [10]. A loss-of-function mutation of *AtSPL9* and *AtSPL15* resulted in altered main stem architecture and enhanced branching [17]. The main inflorescence-changed phenotypes of constitutive expressed *AfSPL14* in *Arabidopsis* and loss-of-function *AtSPL9*, *AtSPL10,* and *AtSPL15* mutants appeared to be similar.

Certain SPLs can be regulated by miR156, two miRNAs that can regulate the expression of SPL proteins at the post-transcriptional level [57]. Similar to *OsSPL14*, *AfSPL14* also had a miR156 cleavage site in its CDS sequence (Figure 3). Because of a point mutation in the OsmiR156-directed site of *OsSPL14*, grain yield was enhanced [25,26]. Many SPLs positively regulate grain yield [23,58,59,60]. The thicker main inflorescence phenotype in *AfSPL14*-constitutive expressed *Arabidopsis* implied that this gene might act positively in the regulation of flower stalk diameter in *A. fasciata*. Further investigation should focus on the morphological changes of flowers in *AfSPL14*-overexpressed and/or *AfSPL14*-silenced *A. fasciata*, the morphological changes of flowers in *AfSPL14*-overexpressed and/or *AfSPL14*-silenced pineapple, and the cloning and functional characterization of possible homologs of *AfSPL14* in pineapple.

## 4. Materials and Methods

### 4.1. Plant Materials and Sample Preparation

The *A. fasciata* specimens used in this study were planted in a greenhouse (ambient temperature of 30–32 °C) located in the experimental area of the Institute of Tropical Crop Genetic Resources, Chinese Academy of Tropical Agricultural Sciences (CATAS). For the tissue-specific expression and western blot analyses, different tissue samples, including mature leaves, central leaves, stems, roots, and various flower organs, were collected.

The wild-type (WT) and transgenic plants of *Arabidopsis* used in this study were of the Columbia ecotype (Col-0). Seeds were surface sterilized in 0.1% HgCl_2_ for 10 min and then washed with sterilized distilled water five times. The washed seeds were then plated on MS medium containing sugar (2%) and agar (0.8%) and incubated in the dark at 4 °C for 2 days. The plates were then moved to a chamber at 23 °C under LD (16 h light) conditions, with a photon flux density (120 µmol m^−2^ s^−1^) for continuous growth.

### 4.2. Isolation and Sequencing of the AfSPL14 Gene

Total RNA was extracted from the central leaves of *A. fasciata* using the hexadecyl trimethyl ammonium bromide (CTAB) method [33], and then used for the RACE at the 5′ and 3′ ends according to the manufacturer’s instructions for the SMARTer™ RACE cDNA Amplification Kit (Clontech, Tokyo, Japan). The specific 5′ and 3′ fragments were cloned into pEASY-blunt vectors (Transgen, Beijing, China), and then sequenced by Thermo Fisher Scientific (Guangzhou, China). The gene accession number of *AfSPL14* is MF114304. The primers used here are listed in Table S3 online.

### 4.3. Bioinformatic Analysis

The ORF of *AfSPL14* was predicted using the ORF Finder (https://www.ncbi.nlm.nih.gov/orffinder/). The sequence logo was generated by the online WebLogo 3 platform (http://weblogo.threeplusone.com/). A phylogenetic tree was constructed with MEGA version 6.0 using the neighbor-joining method with 1000 bootstrap replications [61]. The scheme of exon-intron structures was generated by Gene Structure Display Server 2.0 (http://gsds.cbi.pku.edu.cn/index.php). Putative motifs of variable SPLs were identified by MEME software online with default settings (http://meme-suite.org/tools/meme).

### 4.4. 5′ RLM-RACE

5′ RLM-RACE was performed according to the manufacturer’s instructions of FirstChoice^®^ RLM-RACE Kit (Thermo Fisher Scientific, New York, NY, USA). For the next amplification, 10 μg of total RNA, which was isolated from central leaves of 12-month-old *A. fasciata* plants using the CTAB method [33], was used. The gene specific primers of *AfSPL14* for the first and second PCR products are *AfSPL14*-5outer and *AfSPL14*-5inner, respectively. The second PCR products were gel purified and subcloned into pEASY-T3 Vector (Transgen, Beijing, China) for sequencing. Primers used for 5′ modified RACE are listed in Table S3 online.

### 4.5. RT-qPCR

First-strand cDNA was synthesized using the TransScript One-Step gDNA Removal and cDNA Synthesis SuperMix (Transgen, Beijing, China) according to the manufacturer’s instructions. Quantitative real-time PCR (qPCR) was conducted on a Therma PikoReal 96™ Real-Time PCR System (Thermo Fisher Scientific, Waltham, MA, USA) using the TransStart Tip Green qPCR SuperMix Kit (Transgen, Beijing, China). The total reactions (20 μL) described in this protocol converted total RNA (500 ng~5 μg) into the first-strand cDNA. The first-strand reaction products (20 μL) were diluted with sterilized distilled H_2_O 5 times, and diluted products (1 μL) were used for total qPCR reactions (10 μL). Three biological replicates and three technical replicates were performed. The relative expression levels of specific genes were calculated using the 2^−∆∆Ct^ method with the *β-actin* gene (*ACTB*) of *A. fasciata* or *Arabidopsis* as the internal control [62]. All primers used for qPCR are listed in Table S3 online.

### 4.6. Transgenic Plants

For the transgenic constructs, the coding sequence (CDS) of *AfSPL14* was cloned into the KpnI-SalI sites of the binary vector Cam35S-gfp under the control of the cauliflower mosaic virus (CaMV) 35S promoter. The constructs were then delivered into *Agrobacterium tumefaciens* strain EHA105 by the freeze-thaw method [63]. Col-0 background *Arabidopsis* was transformed using the floral dipping method [64]. For the selection of transgenic plants, the seeds were planted on MS agar medium supplemented with hygromycin (25 mg/L). Seedlings conferring resistance to hygromycin were then transplanted in a chamber at 23 °C under LD conditions. Transgenic plants were verified by genomic PCR and RT-PCR using primers *AfSPL14-OX* F and *AfSPL14-OX* R, which were listed in Table S3 online. T3 transgenic plants were used for next experiments.

### 4.7. Transactivation Analysis of AfSPL14 in Yeast Cells

The yeast strain Y2HGold was transformed with plasmids containing the pGBKT7 (pBD) vector with the ORF or fragments of AfSPL14 fused in frame with GAL4 DNA binding domain. The primers used are listed in Table S3 online. pBD and pGAL4 were used as negative and positive controls, respectively. Transformants were selected on synthetic dropout (SD) medium lacking tryptophan (SD/−Trp) (Clontech, Tokyo, Japan) and then dripped onto SD/−Trp/+AbA/+X-α-gal to determine the transactivation activity.

### 4.8. Exogenous Ethephon Treatment of A. fasciata

To test the response to ethylene, adult (12-month-old) *A. fasciata* plants which were grown in pots in our greenhouse were treated with ethephon (10 mL) at 0.3 g·L^−1^, 0.6 g·L^−1^, 1.2 g·L^−1^, 2.4 g·L^−1^, 4.8 g·L^−1^ for 1, 2, 4, 8, 24, or 48 h. All treatments were applied by pouring the specific concentration of ethephon solution into the leaf whorl of each plant, with the same quantity of water as control. The central leaves were then physically isolated and immediately frozen in liquid nitrogen for further research.

### 4.9. SDS-PAGE and Immunoblot Analysis

Total proteins were extracted from the central leaves of adult *A. fasciata* plants. The physically isolated and immediately frozen central leaves (0.5 g) were homogenized with extraction buffer (1 mL) (Tris (1 mol/L, pH 6.8); DL-dithiothreitol (0.2 mol/L); sodium dodecyl sulfate (4% (g/mL)); glycerol (20%)) by using pestle and mortar. After centrifugation at 12,000 rotation per minute (rpm) for 15 min at 4 °C, the supernatants were transferred into new tubes and 4 times volume of acetones were added. After being vortexed for 2 min and then placed on ice for 1 h, the mixture was centrifuged again, as above. The supernatants were discarded and 4 times volume of acetone was added. The mixture was then vortexed and centrifuged again; the extracted proteins were diluted with 0.5× extraction buffer, boiled at 100 °C for 10 min, and then centrifuged at 13,000 rpm for 5 min. The supernatants (total central leaf proteins) were separated using 15% sodium dodecyl sulfate polyacrylamide gel electrophoresis (SDS-PAGE) containing urea (6 mol/L). After electrophoresis, the proteins were transferred onto nitrocellulose membranes (Amersham Biosciences, Pittsburgh, PA, USA) and probed with a rabbit polyclonal AfSPL14 antibody (Jiaxuan Biotech, Beijing, China) or a rabbit polyclonal Actin antibody (Agrisera, Vännäs, Sweden). After incubation with horseradish peroxidase conjugated goat anti-rabbit IgG (Jiaxuan Biotech, Beijing, China), the signals were detected by enhanced chemiluminescence (Jiaxuan Biotech, Beijing, China). X-ray films were scanned and analyzed using ImageMaster™ 2D Platinum software (GE Healthcare, Pittsburgh, PA, USA). Protein concentration of each extract was determined by using a protein assay kit (Bio-Rad, Hercules, CA, USA) with BSA as the standard.

### 4.10. Data Analysis

Values represent means ± standard deviation of two or three biological replicates. ANOVA was conducted, and the means were separated by Duncan’s New Multiple Range Test (DNMRT).

## Supplementary Materials

Supplementary materials can be found at http://www.mdpi.com/1422-0067/19/7/2085/s1.

## Abbreviations

SBP	SQUAMOSA PROMOTER BINDING PROTEIN
SPL	SBP-LIKE
miRNA	microRNA
*TGA1*	*TEOSINTE GLUME ARCHITECTURE*
LD	long day
RACE	Rapid amplification of cDNA ends
UTR	Untranslated region
ORF	Open reading frame
MW	Molecular weight
pI	Isoelectric point
Jp	Joint peptide
NLS	Nuclear localization signal
RT-qPCR	Reverse transcription followed by quantitative real-time PCR
RLM-RACE	RNA ligase mediated rapid amplification of cDNA ends
DAF	Day after flowering
WT	Wild Type
*SOC1*	*SUPPRESSOR OF OVEREXPRESSION OF CONSTANS 1*
*FUL*	*FRUITFULL*
*AP1*	*APETALA1*
*LFY*	*LEAFY*
*FT*	*Flowering Locus T*
*AP2*	*APETALA2*
*AP3*	*APETALA3*
CAM	Crassulacean acid metabolism
EIN3	ETHYLENE INSENSITIVE 3
GA	Gibberellic acid
CTAB	Hexadecyl trimethyl ammonium bromide
CDS	the coding sequence
SDS-PAGE	Sodium dodecyl sulfate polyacrylamide gel electrophoresis
