# Supplementary material for: Constitutive Expression of Aechmea fasciata SPL14 (AfSPL14) Accelerates Flowering and Changes the Plant Architecture in Arabidopsis
Source: Int J Mol Sci. 2018 Jul 18;19(7):2085. doi: 10.3390/ijms19072085 (PMC6073119; doi:10.3390/ijms19072085)
Supplement: Supplementary file 1 [file ijms-19-02085-s001.pdf]

International Journal of
Molecular Sciences

MDPI

Supplementary Information

Constitutive expression of *Aechmea fasciata SPL14* (*AfSPL14*) accelerates flowering and changes the plant architecture in *Arabidopsis*

Ming Lei [1,2,3,4], Zhi-ying Li [1,2,3,4], Jia-bin Wang [1,2,3,4], Yun-liu Fu [1,2,3,4], Meng-fei Ao [1,2,3,4] and Li Xu [1,2,3,4]*

* Correspondence: xllzy@263.net; Tel.: +86-898-2330-0284

Table S1. The sequences of SBP domains and accession numbers of selected plant SPL proteins for phylogenetic analysis.

Protein Name	Sequences of SBP domains	Accession No
AtSPL1	CQVENCEADLSKVKDYHRRHKVCEMHSKATSATVGGIL<br>QRFCQQCSRFHLLQEFDEGKRSCRRRLAGHNKRRRK	AT2G47070
AtSPL2	CQVEGCNLDLSSAKDYHRKHRICENHSKFPKVVVSGVER<br>RFCQQCSRFHCLSEFDEKKRSCRRRLSDHNARRRK	AT5G43270
AtSPL3	CQVESCTADMSKAKQYHKRHKVCQFHAKAPHVRISG-<br>LHQRFCQQCSRFHALSEFDEAKRSCRRRLAGHNERRRK	AT2G33810
AtSPL4	CQVDRCTADMKEAKLYHRRHKVCEVHAKASSVFLSGLN<br>QRFCQQCSRFHDLQEFDEAKRSCRRRLAGHNERRRK	AT1G53160
AtSPL5	CQVDRCTVNLTEAKQYYRRHRVCEVHAKASAATVAGV<br>RQRFCQQCSRFHELPEFDEAKRSCRRRLAGHNERRRK	AT3G15270
AtSPL6	CQVYGCSKDLSSSKDYHKRHRVCEAHSKTSVVIVNGLEQ<br>RFCQQCSRFHFLSEFDDGKRSCRRRLAGHNERRRK	AT1G69170
AtSPL7	CQVPDCEADISELKGYHKRHRVCLRCATASFVVLDGENK<br>RYCQQCGKFHLLPDFDEGKRSCRRKLERHNNRRKR	AT5G18830
AtSPL8	CQAEGCNADLSHAKHYHRRHKVCEFHSKASTVVAAGL<br>SQRFCQQCSRFHLLSEFDNGKRSCRKRLADHNRRRRK	AT1G02065
AtSPL9	CQVEGCGMDLTNAKGYYSRHRVCGVHSKTPKVTVAGIE<br>QRFCQQCSRFHQLPEFDLEKRSCRRRLAGHNERRRK	AT2G42200
AtSPL10	CQIDGCELDLSSSKDYHRKHRVCETHSKCPKVVVSGLER<br>RFCQQCSRFHAVSEFDEKKRSCRKRLSHHNARRRK	AT1G27370
AtSPL11	CQIDGCELDLSSAKGYHRKHKVCEKHSKCPKVSVSGLER<br>RFCQQCSRFHAVSEFDEKKRSCRKRLSHHNARRRK	AT1G27360
AtSPL12	CQVDNCGADLSKVKDYHRRHKVCEIHSKATTALVGGIM<br>QRFCQQCSRFHVLEEFDEGKRSCRRRLAGHNKRRRK	AT3G60030
AtSPL13	CLVDGCDSDFSNCREYHKRHKVCDVHSKTPVVTINGHK<br>QRFCQQCSRFHALEEFDEGKRSCRKRLDGHNRRRRK	AT5G50570
AtSPL14	CQVDNCTEDLSHAKDYHRRHKVCEVHSKATKALVGKQ<br>MQRFCQQCSRFHLLSEFDEGKRSCRRRLAGHNRRRRK	AT1G20980
AtSPL15	CQVEGCRMDLSNVKAYYSRHKVCCIHSKSSKVIVSG-<br>LHQRFCQQCSRFHQLSEFDLEKRSCRRRLACHNERRRK	AT3G57920
AtSPL16	CQVDNCKEDLSIAKDYHRRHKVCEVHSKATKALVGKQ<br>MQRFCQQCSRFHLLSEFDEGKRSCRRRLDGHNRRRRK	NP_177784.6
OsSPL1	CQVDGCTVNLSSARDYNKRHKVCEVHTKSGVVRIKNVE<br>HRFCQQCSRFHFLQEFDEGKKSCRSRLAQHNRRRRK	XP_015614279.1
OsSPL2	CSVEGCAADLSKCVRDYHRRHKVCEAHSKTAVVTVAGQ<br>QQRFCQQCSRFHLLGEFDEEKRSCRKRLDGHNKRRRK	XP_015611358.1
OsSPL3	CQVEGCNVDLSSAKPYHRKHRVCEPHSKTLKVIVAGLER<br>RFCQQCSRFHGLAEFDQKKRSCRRRLHDHNARRRK	XP_015626884.1
OsSPL4	CQVEGCGVELVGVKDYHRKHRVCEAHSKFPRVVVAGQ<br>ERRFCQQCSRFHALSEFDQKKRSCRRRLYDHNARRRK	XP_015623075.1

OsSPL5	CQAEGCKADLSAAKHYHRRHKVCDFHAKAAAVLAAG KQQRFCQQCSRFHVLAEFDEAKRSCRKRLTEHNRRRRK	XP_015624330.1
OsSPL6	CQVEGCTADLTGVRDYHRRHKVCEMHAKATTAVVGNT VQRFCQQCSRFHPLQEFDEGKRSCRRRLAGHNRRRRK	XP_015631511.1
OsSPL7	CQVEGCDITLQGVKEYHRRHKVCEVHAKAPRVVVHGTE QRFCQQCSRFHVLAEFDDAKKSCRRRLAGHNERRRR	XP_015635344.1
OsSPL8	CQAEGCKADLSSAKRYHRRHKVCEHHSKAPVVVTAGGL HQRFCQQCSRFHLLDEFDDAKKSCRKRLADHNRRRRK	XP_015634037.1
OsSPL9	CQVPGCEADIRELKGYHRRHRVCLRCAHAAAVMLDGV QKRYCQQCGKFHILLDFDEDKRSCRRKLERHNKRRRR	XP_015640052.1
OsSPL10	CQAEGCKADLSGAKHYHRRHKVCEYHAKASVVAASGK QQRFCQQCSRFHVLTEFDEAKRSCRKRLAEHNRRRRK	XP_015642406.1
OsSPL11	CQVEGCGLELGGYKEYYRKHRVCEPHTKCLRVVVAGQD RRFCQQCSRFHAPSEFDQEKRSCRRRLSDHNARRRK	XP_015641499.1
OsSPL12	CQVEGCKVDLSSAREYHRKHKVCEAHSKAPKVIVSGLER RFCQQCSRFHGLAEFDQKKKSCRRRLSDHNARRRK	XP_015643462.1
OsSPL13	CQVERCGVDLSEAGRYNRRHKVCQTHSKEPVVLVAGLR QRFCQQCSRFHELTEFDDAKRSCRRRLAGHNERRRK	XP_015645415.1
OsSPL14	CQVEGCGADLSGIKNYYCRHKVCFMHSKAPRVVVAGLE QRFCQQCSRFHLLPEFDQGKRSCRRRLAGHNERRRR	XP_015650813.1
OsSPL15	CQVDDCRADLTNAKDYHRRHKVCEIHGKTTKALVGNQ MQRFCQQCSRFHPLSEFDEGKRSCRRRLAGHNRRRRK	XP_015649921.1
OsSPL16	CAVDGCKEDLSKCRDYHRRHKVCEAHSKTPLVVVSGRE MRFCQQCSRFHLLQEFDEAKRSCRKRLDGHNRRRRK	XP_015649377.1
OsSPL17	GGSGGGGGGGGGDDVHGRHKVCYMHAKEPIVVVAG LEQRFCQQCSRFHQLPEFDQEKKSCRRRLAGHNERRRK	XP_015610961.1
OsSPL18	CAVDGCKADLSKHRDYHRRHKVCEPHSKTPVVVVSG-REMRFCQQCSRFHLLGEFDEAKRSCRKRLDGHNRRRRK	XP_015610873.1
OsSPL19	CSVDGCRSDLSRCRDYHRRHKVCEAHAKTPVVVVAGQE QRFCQQCSRFHNLAEFDDGKKSCRKRLDGHNRRRRK	XP_015617950.1
PpSBP1	CQAEGCKDDLSNAKHYHRRHKVCELHSKAPTVTVGGH TQRFCQQCSRFHHLGEFDEGKRSCRKRLADHNRRRRK	AJ968320
PpSBP2	CQVDGCTADLSRAKDYHRRHKVCEAHSKAPTTLVSRVR QRFCQQCSRFHPLDKFDEDKRSCRRRLAGHNKRRRK	CAI91313.1
PpSBP3	CQVQGCDADLSCCKDYHKRHKVCEMHSKAATAIAAGI EQRFCQQCSRFHVLKEFDEGKRSCRRRLAGHNQRRRK	CAI91301.1
PpSBP4	CQAEGCKTDLSTSKQYHRRHKVCELHSKAPNVQVGGQT QRFCQQCSRFHSLEEFDNGKRSCRKRLADHNRRRRK	AJ968319
PpSBP5	CQVPACGADLAGLKGYHQRHRVCLQCANSTTVILRDIP HRYCQQCGKFHVLSDFDEGKRSCRFKLERHNNRRRRK	ABM67299.1
PpSBP6	CQVEGCKADLSGCKDYHKRHKVCEMHSKAPKCIAAGIE QRFCQQCSRFHVLTEFDEGKRSCRRRLAGHNERRRK	ABM67300.1
PpSBP7	CQAEGCKSDLSTAKQYHRRHKVCELHSKAPNVVAGGQT QRFCQQCSRFHSLGEFDDGKRSCRKRLADHNRRRRK	ABM67301.1
PpSBP8	CQAEGCKFDLSLAKPYHRRHKVCELHSKAPNVIAGGQT QRFCQQCSRFHSLGEFDDGKRSCRKRLADHNRRRRK	XP_001775629.1
PpSBP9	CQAEGCKADLNVTKNYYRRHKVCEFHSKTPIVIVGGHT QRFCQQCSRFHRLGEFDDGKRSCRKRLADHNRRRRK	ABM67302.1
PpSBP10	CQVDGCTADLSKAKDYHRRHKVCETHSKASTAQVSRVT QRFCQQCSRFHALDQFDEGKRSCRRRLAGHNKRRRK	ABM67303.1
PpSBP11	CQVDACKADLSKAKDYYRRHKVCETHSKATKAPVSRLM QRFCQQCSRFHPLQEFDEGKRSCRRRLAGHNRRRRK	ABV03806.1
PpSBP12	CQAEGCKADLSQAKQYHRRHKVCEHHSKALNVVANG QTQRFCQQCSRFHLLGEFDDGKRSCRKRLADHNRRRRK	ABM67304.1
PpSBP13	CQVEGCKADLSGCKDYHKRHKVCEMHSKAPKCIAAGIE QRFCQQCSRFHVLTEFDEGKRSCRRRLAGHNERRRK	ABM67305.1

Table S2. The consensus sequences of the putative motifs of variable SPLs in group III identified by MEME software online (http://meme-suite.org/tools/meme).

Motif name	Sequences
Motif 1	YYCRHKVCYMHSKAPRVVVAGLEQRFCQQCSRFHQLPEFDQEKRSCRRRL
Motif 2	RCQVEGCGVDLSGVK
Motif 3	GLKFGKKIYFED
Motif 4	ATDSSCALSLLSTQPWDHTT
Motif 5	AGHNERRRKPQ
Motif 6	PLSSRYGRJAPSLHEEPNRFR
Motif 7	FVLDFSYPRVPSSVRDAWPAIQPGDRISGGIQWQGGVEPHGHRSAVAGY
Motif 8	FDHSSHHMNWSL
Motif 9	EPPPGQIHNGHFSGELELALQ
Motif 10	QYMEDENT

Table S3. Primers used in this study.

Name	Sequences (from 5′ to 3′)
AfSPL14 5′ RACE GSP1	GCG GCA TTA CTG GAG TTC GGT T
AfSPL14 5′ RACE GSP2	GGT GGC GAC TCC TGA GAA GCA TT
AfSPL14 3′ RACE GSP1	GTA GGA AAC CAC CTG TCC CTT TGT CA
AfSPL14 3′ RACE GSP2	GAT TCG CCT CCG CTG CCC CTA A
AfSPL14 cDNA F	CTC TCT CCC TCT CTC TGG GGT GTC T
AfSPL14 cDNA R	GCT ATA GTT GGT CAT GAT CAC ATT A
AfSPL14-5outer	GGC ATT TCA TGT GAA CTG GGT C
AfSPL14-5inner	CGG CAT TAC TGG AGT TCG GTT A
AfSPL14-pBD F	CGG AAT TCA TGG AGA AGG GTT CGG GCT CCG TTG
AfSPL14-pBD R	AAC TGC AGC AGA GAC CAG TGC ATG CCG TGA
AfSPL14N-pBD F	GGA ATT CAT GGA GAA GGG TTC GGG CTC
AfSPL14N-pBD R	ACG CGT CGA CTG GTT TCC TAC GAC GCT CG
AfSPL14C-pBD F	GGA ATT CCC TGT CCC TTT GTC ATC TCG
AfSPL14C-pBD R	ACG CGT CGA CCT ACA GAG ACC AGT GCA TGC
AfSPL14-OX F	GGG GTA CCA TGG AGA AGG GTT CGG GCT CCG TTG
AfSPL14-OX R	GCG TCG ACC TAC AGA GAC CAG TGC ATG CCG TGA
AfSPL14 qRT-PCR F	CTT CTT CTC ACC CAC GGA ACT
AfSPL14 qRT-PCR R	ACA TGG CTA TGC GGC ATT AC
AfACTB qRT-PCR F	TAC AGT GTC TGG ATT GGG GG
AfACTB qRT-PCR R	CGG ATT CAT CAT ACT CAC CCT T
AtLFY qRT-PCR F	CGA GCA CGC TTG TGG GTA T
AtLFY qRT-PCR R	TTG CAA TCG TCT CCG TTC AG
AtAP1 qRT-PCR F	TGG GTG GTC TGT ATC AAG AAG ATG
AtAP1 qRT-PCR R	CCA AGG TTG CAG TTG TAA ACG
AtAP2 qRT-PCR F	GGT GTT GCT TCT GGC TTT CC
AtAP2 qRT-PCR R	GGT CCA CGC CGA CTC TTT T
AtAP3 qRT-PCR F	GGA GAT TAC GAC TCA GTT CTT GGA T
AtAP3 qRT-PCR R	GTG GTG ATG GTT CTG GTG GA
AtFUL qRT-PCR F	CAA CTT GTT GGC CGA GAC G
AtFUL qRT-PCR R	TGG AGC GCA GAT ATG GAT TC
AtSOC1 qRT-PCR F	CTA AAC GTA AAC TCT TGG GA
AtSOC1 qRT-PCR R	CAG AAC TTG GGC TAC TCT CT
AtFT qRT-PCR F	GGTGGAGAAGACCTCAGGAACT
AtFT qRT-PCR R	TGCCAAGCTGTCGAAACAATA
AtACTB qRT-PCR F	TGT GCC AAT CTA CGA GGG TTT
AtACTB qRT-PCR R	TTT CCC GCT CTG CTG TTG T

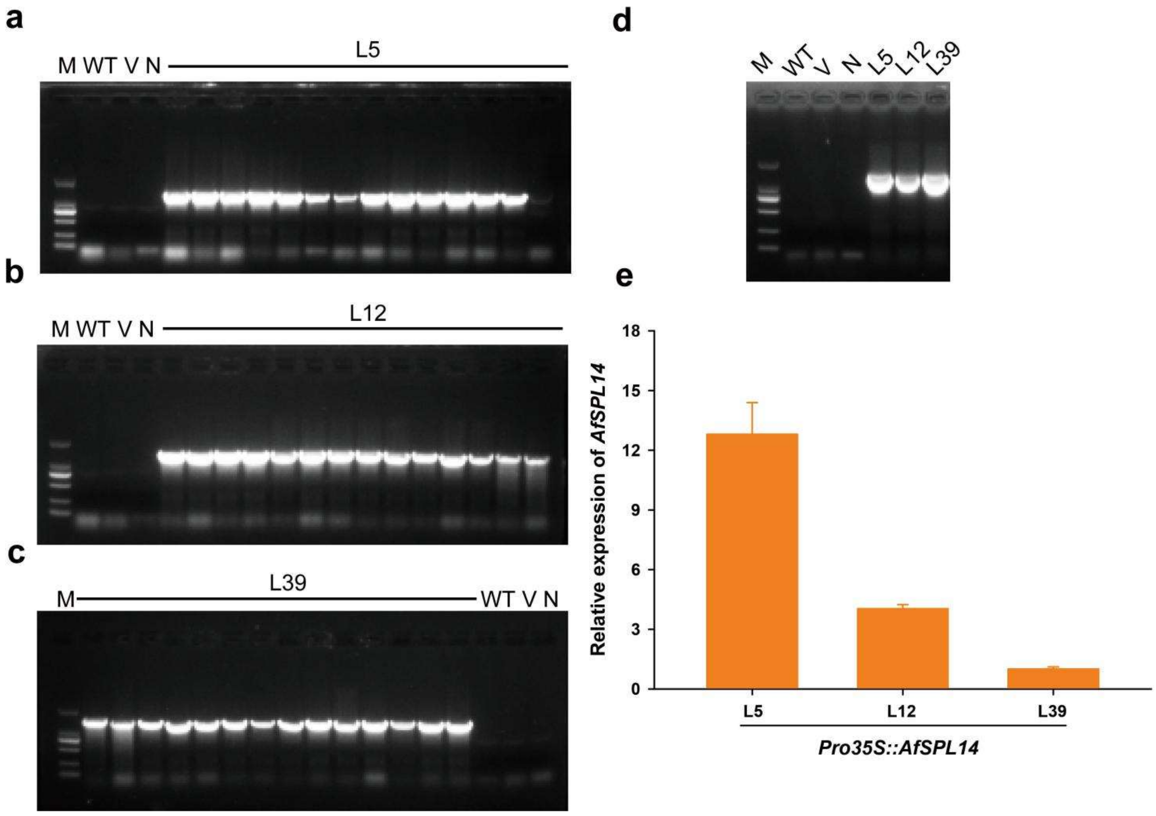

Figure S1 Verification of *Pro35S::AfSPL14* transgenic plants. DNA of randomly selected T3 plants of variable lines (L5, L12, L39) were extracted and used for PCR verification using *AfSPL14-OX* F and *AfSPL14-OX* R primers (**a, b, c**). Furthermore, RNA of variable transgenic lines were also extracted, and PCR (**d**) or RT-qPCR (**e**) were conducted. M: DNA Ladder 2000; WT: the Wild Type; V: Vector; N: negative control which used double distilled H_2O (ddH_2O) as templates in PCR.

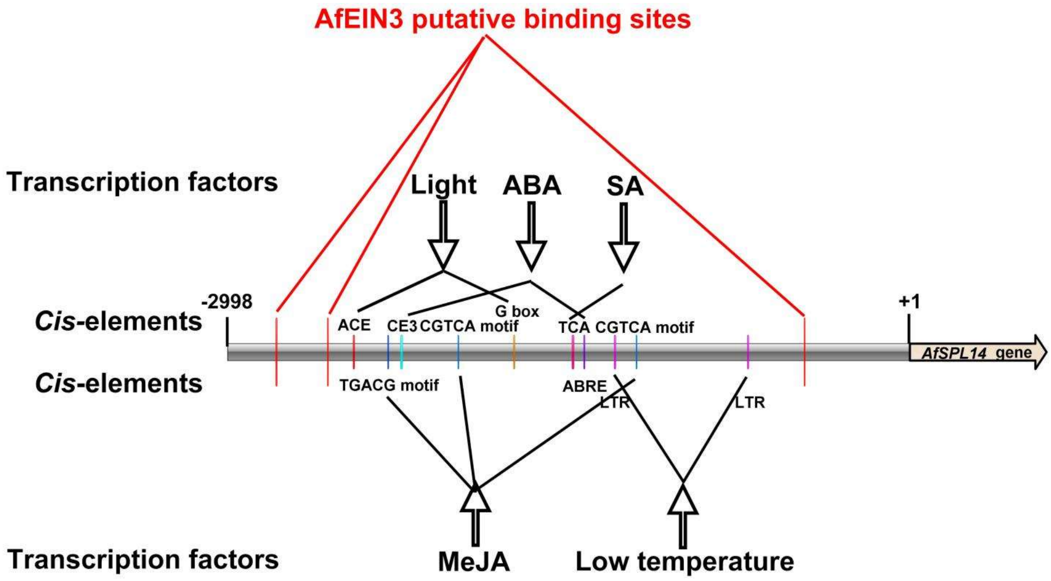

Figure S2 Representative cis-elements enclosed in the nearly 3000-bp-length promoter sequence of *AfSPL14*. Amounts of cis-elements, which might response to light, abscisic acid (ABA), salicylic acid (SA), methyl jasmonate (MeJA), low temperature, etc, were within the promoter. Three 5′-ATGTA-3′ core sequences, which might interact with ETHYLENE INSENSITIVE 3 (EIN3), a crucial factor in ethylene signaling pathway that could activate or inhibit the expression of downstream genes in transcriptional level, were also within the promoter.
